# Molecular landscape of kidney allograft tissues data integration portal (NephroDIP): a curated database to improve integration of high-throughput kidney transplant datasets

**DOI:** 10.3389/fimmu.2024.1469500

**Published:** 2024-09-27

**Authors:** Alex Boshart, Stefan Petrovic, Mark Abovsky, Chiara Pastrello, Sofia Farkona, Kieran Manion, Slaghaniya Neupane, Maya Allen, Igor Jurisica, Ana Konvalinka

**Affiliations:** ^1^ Toronto General Hospital Research Institute, University Health Network, Toronto, ON, Canada; ^2^ Ajmera Transplant Centre, University Health Network, Toronto, ON, Canada; ^3^ Institute of Medical Science, University of Toronto, Toronto, ON, Canada; ^4^ Osteoarthritis Research Program, Division of Orthopedic Surgery, Schroeder Arthritis Institute, University Health Network, Toronto, ON, Canada; ^5^ Data Science Discovery Centre for Chronic Diseases, Krembil Research Institute, University Health Network, Toronto, ON, Canada; ^6^ Department of Laboratory Medicine and Pathobiology, University of Toronto, Toronto, ON, Canada; ^7^ Departments of Medical Biophysics and Computer Science, and Faculty of Dentistry, University of Toronto, Toronto, ON, Canada; ^8^ Institute of Neuroimmunology, Slovak Academy of Sciences, Bratislava, Slovakia; ^9^ Department of Medicine, Division of Nephrology, University Health Network, Toronto, ON, Canada

**Keywords:** data integration, integrative computational biology, kidney transplantation, high-throughput data, antibody-mediated rejection, interstitial fibrosis and tubular atrophy, transplant immunosuppression, LGALS1

## Abstract

**Introduction:**

Kidney transplantation is the optimal treatment for end-stage kidney disease; however, premature allograft loss remains a serious issue. While many high-throughput omics studies have analyzed patient allograft biospecimens, integration of these datasets is challenging, which represents a considerable barrier to advancing our understanding of the mechanisms of allograft loss.

**Methods:**

To facilitate integration, we have created a curated database containing all open-access high-throughput datasets from human kidney transplant studies, termed NephroDIP (Nephrology Data Integration Portal). PubMed was searched for high-throughput transcriptomic, proteomic, single nucleotide variant, metabolomic, and epigenomic studies in kidney transplantation, which yielded 9,964 studies.

**Results:**

From these, 134 studies with available data detailing 260 comparisons and 83,262 molecules were included in NephroDIP v1.0. To illustrate the capabilities of NephroDIP, we have used the database to identify common gene, protein, and microRNA networks that are disrupted in patients with chronic antibody-mediated rejection, the most important cause of late allograft loss. We have also explored the role of an immunomodulatory protein galectin-1 (LGALS1), along with its interactors and transcriptional regulators, in kidney allograft injury. We highlight the pathways enriched among LGALS1 interactors and transcriptional regulators in kidney fibrosis and during immunosuppression.

**Discussion:**

NephroDIP is an open access data portal that facilitates data visualization and will help provide new insights into existing kidney transplant data through integration of distinct studies and modules (https://ophid.utoronto.ca/NephroDIP).

## Introduction

Kidney transplantation is the best treatment for end-stage kidney disease (1); however, approximately 50% of kidney transplant recipients lose their graft by 10 years post-transplant (2). High-throughput studies are increasingly conducted on patient-derived allograft biospecimens to better understand the molecular mechanisms of kidney allograft disease that leads to premature allograft loss, with the goal of developing precision medicine. The datasets generated by these studies represent a significant resource, as integrating them could enable scientists to derive novel observations or hypotheses about the molecular signatures and regulatory networks underpinning allograft loss. However, the high-throughput data from different studies are often challenging to integrate due to their heterogeneity, including distinct methods used to generate data, molecules evaluated by the study (e.g., mRNA vs microRNA vs protein), patient populations, and underlying clinical questions. Platforms that curate and integrate the molecular and clinical data from all available relevant studies may uniquely enable researchers to perform analyses that would otherwise be difficult or impossible.

Several prior studies have started to address this need. Nephroseq is a publicly available platform created by the University of Michigan to help researchers assess gene expression in the human kidney (https://www.nephroseq.org/resource/login.html). This database is primarily focused on native kidney disease and only contains mRNA expression, precluding comparison of multi-omics datasets. Renal Gene Expression Database (RGED) is another platform for researchers to assess kidney gene expression. This database is relatively small and limited to the transcriptome of certain renal diseases (3). Data integration portals have been created with aggregated data from diseases such as osteoarthritis (OsteoDIP) (4, 5) or liver cancer (CDIPLiver) (6, 7). However, such curated platforms on a multi-omics scale in the context of kidney transplantation are limited, despite a large number of high-throughput molecular studies published.

In this manuscript, we describe our construction of a curated and annotated database called NephroDIP (https://ophid.utoronto.ca/NephroDIP), which integrates all high-throughput datasets extracted from peer-reviewed, published manuscripts relevant to the kidney allograft. NephroDIP is a Data Integration Portal (DIP) that contains data on mRNA, proteins, metabolites, epigenetic signatures, as well as single nucleotide variants (SNV). We show, using several examples, how NephroDIP can be used to leverage these publicly available datasets to generate new insights into the molecular underpinnings of various kidney transplant phenotypes. Lastly, we have made NephroDIP an open access resource for other scientists interested in analysing high-throughput datasets across multiple molecule types in the kidney allograft.

## Materials and methods

### Data collection

We performed a search in PubMed for high-throughput transcriptomic, proteomic, SNV, metabolomic, and epigenomic studies on kidney transplantation. Each search was performed by filtering for articles that were published from 2002 onwards (as most high-throughput studies were published after this point), had text in English, analyzed human samples, and had full text availability. The PubMed search terms were as follows:

Transcriptome: ((kidney transplant) OR (kidney allograft)) AND ((gene expression) OR (microarray) OR (RNA sequencing)).

Proteome: ((kidney transplant) OR (kidney allograft)) AND ((protein expression) OR (proteomic analysis)).

SNV: ((kidney transplant) OR (kidney allograft)) AND ((single nucleotide polymorphism) OR (single nucleotide variant)).

Metabolome: ((kidney transplant) OR (kidney allograft)) AND ((metabolome) OR (metabolite)).

Epigenome: ((kidney transplant) OR (kidney allograft)) AND ((methylation) OR (epigenome) OR (epigenetic) OR (acetylation)). These searches captured papers available on PubMed by the cutoff date of May 31, 2023.

### Inclusion and exclusion criteria

Included papers were those that contained original data generated using high-throughput methodology (e.g., gene expression studies using microarray technology or RNA sequencing) in a cohort of kidney transplant patients. Papers were excluded from NephroDIP if they were identified as a review (including standard reviews, comments, meta-analyses, editorials, guidelines, and letters to editor); did not analyze kidney transplant patients; addressed unrelated questions (e.g., IHC analysis of various proteins in the transcriptome search); only used low-throughput methodology (e.g., PCR, ELISA, Western Blot); only analyzed animal models or cell lines; did not generate original data (e.g., analysis of downloaded gene expression omnibus (GEO) sets); were retracted; were not written in English; did not include p-values or q-values as measures of significance; solely used a fold change threshold as its measure of significance; or did not have accessible data or downloadable tables containing the high-throughput analyses.

### NephroDIP annotation and upload

Articles identified based on the inclusion criteria were assessed for the presence of data noting molecular identifiers (e.g., gene symbol or UniProt ID) in supplementary materials and tables within the text. Once available data were identified, tables were uploaded to the NephroDIP database. Molecules were annotated as up-regulated or down-regulated between the comparison groups studied in the paper based on the fold change or score reported in the data. For SNVs, reported ratios were annotated as increased or decreased risk of outcome dictated by the publication. This included measures such as hazard ratios (HR) and odds ratios (OR). Some manuscripts used effect size (ES) and beta values (estimated ES). We included these papers and their values within NephroDIP; however, because of the lack of directionality inherent to ES, we used “ES <0” or “ES >0”. The epigenetic search included the gene symbols present, but changes were annotated as hyper- and hypo-methylated where appropriate. None of the identified studies examined acetylation.

Due to reporting differences between studies, gene and methylation information were standardized to gene symbols for identification and searches. Within the gene search, mirbase notation was used for microRNAs while GenBank and Ensembl accession IDs were used for long non-coding RNA (lncRNA) (8, 9). The protein search used gene symbols and UniProtKB identifiers. For studies solely reporting gene symbols, a UniProtKB identifier was not mapped due to the uncertainty in which specific protein was encoded by the gene. However, for studies providing solely UniProtKB identifiers, the “ID mapping” functionality present on UniProt.org was used to map each identifier to its respective gene symbol, where possible. Some UniProt IDs were outdated, and in this case, UniProtKB was used to convert these older identifiers to the updated format that is standardized across NephroDIP. Additionally, some studies included a list of multiple possible UniProtKB identifiers for one peptide sequence found in the data analysis. Only the first UniProt ID was included for each entry in these situations, as the list is ordered by IDs most likely to be the correct match for the detected peptide sequence. The metabolite search used human metabolome database (HMDB; https://hmdb.ca) identifiers (10) and the SNV search used dbSNP notation with gene symbols mapped where possible using the Ensembl VEP tool (9). Some articles did not have these identifiers (e.g., giving metabolite name instead of HMDB code); these were annotated manually by searching the metabolite names in HMDB version 5 and utilizing the HMDB identifiers for standardization within NephroDIP. Many studies did not report regulation or ratio changes in the case of SNVs, and only gave a fold change, log fold change, HR, OR, or other measure. This information was also annotated manually with either up- or down-regulation, increased/decreased ratio, or hyper/hypo-methylated, as relevant.

Additionally, p- and q-values were included in NephroDIP to show the significance or lack of significance for each analyte. If analytes did not meet the threshold of significance set by the authors of the study, they were labeled as “NOT SIGNIFICANT”. Furthermore, if studies did not have specific p-values for their analytes, but provided a threshold of significance, this was used instead. For example, if all genes in a study were said to be significantly differentially expressed between comparator groups with a p-value threshold of 0.05, then all genes had their p-values annotated as “≤0.05” in the corresponding data file. Within NephroDIP, p-value thresholds of ≤0.05, ≤0.01, and ≤0.001 are included as search parameters.

Corresponding files were created for each comparison found in the data files. These files provide details about the study conducted, including the study citation and PubMed ID, a “comparison” tab indicating study groups being compared, and the “outcome/exposure” tab that highlights the specific outcome/exposure(s) of interest in these groups. A master list was created for this “outcome/exposure” tab, including 18 different categories that exist as a search parameter in NephroDIP. The number of case and control samples, and demographic information (i.e. sex and age) were also included. Age was grouped into three categories: paediatric (<18), adult (18–65), and elderly (>65). The type of bio-specimen was annotated (i.e. blood, kidney tissue, and urine). The methodology the authors employed in their study was also reported, with a master list of 15 different methods available as a search parameter in NephroDIP. Comparisons were consolidated to group similar clinical questions and make them easily searchable.

Another search parameter is “data’’, which has two possible selections: “pre-selected” or “not pre-selected”. If the authors only included the top differentially expressed analytes, these data were denoted in NephroDIP as “pre-selected”, as only a portion of the total data is available. A “data reference” tab was also included in the files that highlight the datasets (i.e. public database identifiers, tables, and supplementary data) from the studies that were uploaded to NephroDIP.

### Statistical analysis

For all pathway and gene ontology analyses performed using data extracted from NephroDIP, significance was determined based on a false discovery rate with the Benjamini-Hochberg correction (FDR: BH) of q<0.05 or q<0.01.

## Results

### Descriptive analysis

**Figure 1:**
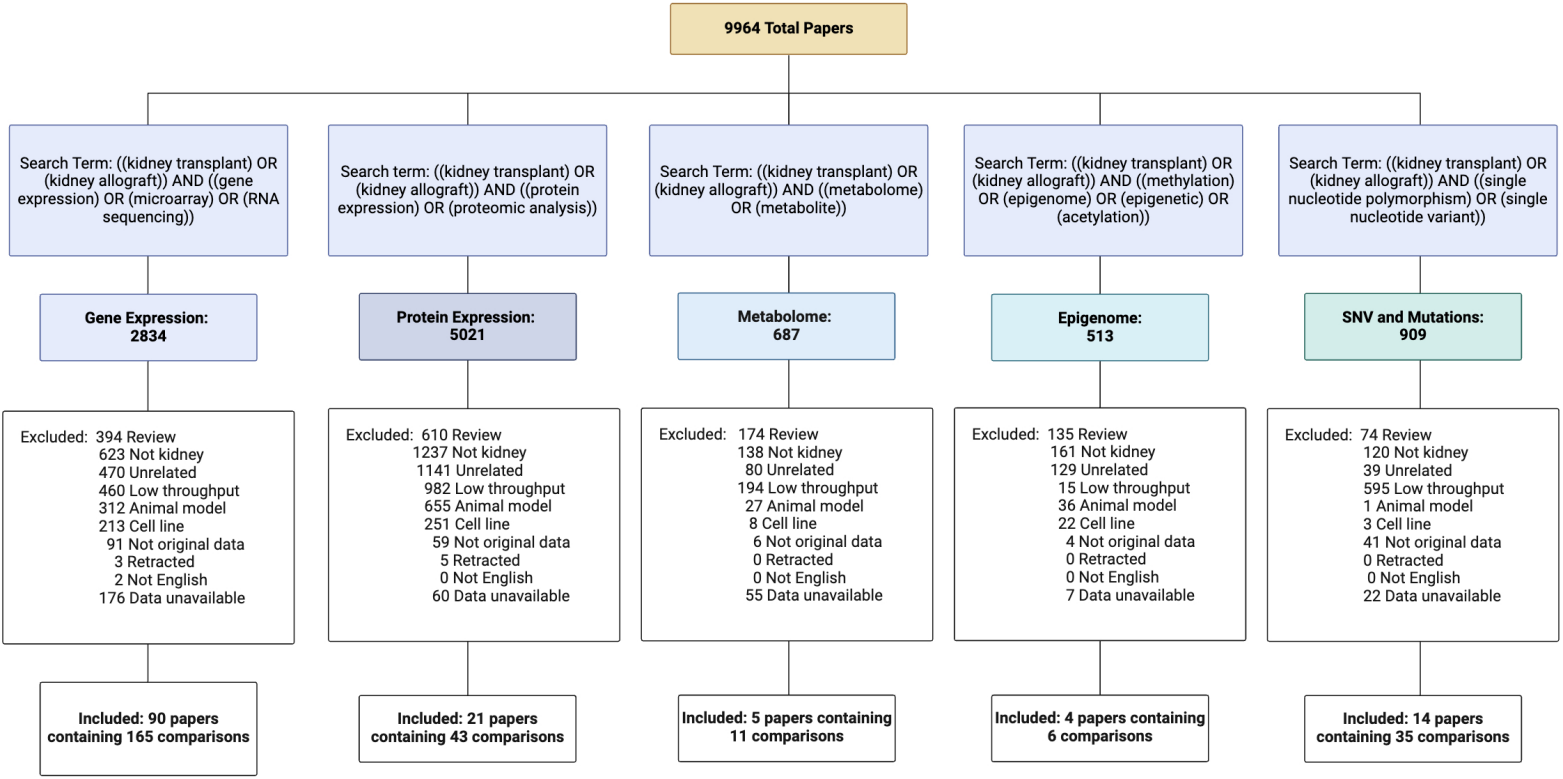
Curation of NephroDIP manuscripts from 5 PubMed searches including transcriptomic, proteomic, metabolomic, epigenomic, and single nucleotide variant (SNV) human kidney transplant studies. Excluded article numbers and the reasons for exclusion are listed along with the final number of studies and comparisons included for each search term. Articles included in NephroDIP v1.0 were those that used high throughput methodology in a cohort of kidney transplant patients, had original data, were written in English, used either p- or q-values as measures of significance, used fold change to see differences between groups of interest (hazard ratios, odds ratios, or effect size were used for SNV studies), and had accessible and downloadable tables that described the comparison between groups.

NephroDIP v1.0 (https://ophid.utoronto.ca/NephroDIP/) contains a total of 134 studies comprising 260 unique comparisons, from an initial PubMed search of 9,964 studies (**Figure 1**). NephroDIP contains data from 83,262 molecules with expression information across three bio-specimens: kidney, blood, and urine. These molecules include 44,832 genes, 4,125 proteins, 28,627 SNVs, 77 metabolites, and 5,601 epigenetic signatures (**Table 1**).

**Table 1:** Expression search statistics including the number of genes, proteins, SNVs, metabolites, and epigenetic regions in the kidney, urine, and blood that were uploaded to NephroDIP.

Bio-specimen	Number of Genes	Number of Proteins	Number of SNVs	Number of Metabolites	Number of Epigenetic Regions
**Kidney**	19,715	2,433	99	0	2,711
**Urine**	7,582	1,049	0	19	0
**Blood**	17,535	643	28,528	58	2,890

With the help of NephroDIP, users can search a molecule of interest and assess its expression across multiple studies, as well as download the data and perform analyses of their own design. Users can also connect their molecular search results to external tools, including Drugst.One, NAViGaTOR, mirDIP, PathDIP, and the Catalogue of Transcriptional Regulatory Interactions (Catrin) (11–14), to better understand regulation and drug targeting in their molecules of interest. Conversely, users can start their search of the database by selecting their disease of interest and perform analyses to investigate the differential expression of several molecules (mRNA, protein, etc.) compared to healthy controls or other disease states.

### Conserved molecular signatures in chronic antibody mediated rejection

Chronic antibody mediated rejection (cABMR) is the most important cause of late kidney allograft loss (15). It presents months to years after transplantation and has diagnostic criteria of chronic glomerulopathy, evidence of antibody interaction with the graft endothelium (e.g., C4d deposition and microvascular inflammation), and detection of donor specific antibodies (DSAs) in the serum (16). Chronic glomerulopathy is a particularly aggressive lesion of cABMR that is associated with poor prognosis, and is characterized by multilayering and duplication of the glomerular and tubular basement membranes (17). Around 50% of cABMR cases present without C4d deposition and circulating DSAs, which makes diagnosis and treatment of cABMR challenging (16). Furthermore, the molecular underpinnings are widely debated, and treatments are lacking. Having the ability to integrate high throughput datasets from patients with cABMR could help inform more specific and conserved molecular mechanisms that can help in understanding the disease pathogenesis.

To illustrate the power of ascertaining and integrating datasets using NephroDIP, we first sought to identify the common signatures of cABMR across published studies with high-throughput data and ask the question: *What are the common signatures of cABMR in the kidney, and are they conserved across the blood and urine?* Searching NephroDIP for ABMR as an outcome/exposure uncovered 19 studies with 31 comparisons as highlighted in **Supplementary Figure S1**. Of these, 4 studies with 5 comparisons analyzed gene and protein expression in cABMR. One comparison analyzed mRNA expression via microarray in peripheral blood mononuclear cells (PBMCs) isolated from 29 patients diagnosed with biopsy-proven cABMR compared to 29 stable graft patients (18). They found 45 genes significantly differentially expressed (p<0.05) between the two groups. Two additional studies analyzed kidney tissue. The first performed single-cell RNA sequencing (scRNAseq) in 2 kidney explants with cABMR, and compared them to 6 patients with tumour-adjacent healthy kidney tissue (19). Comparing these two groups, the authors found 356 genes significantly differentially expressed (q<0.001). The second study evaluating kidney tissue examined glomerular protein expression in 10 kidneys with cABMR via mass spectrometry and compared this protein expression to 8 patients with stable grafts and 11 patients with acute ABMR (aABMR) (20). They found 335 proteins significantly differentially expressed between cABMR and stable grafts (q<0.05), and 137 proteins significantly differentially expressed between cABMR and aABMR (q<0.05). Finally, one study analyzed proteins in urine extracellular vesicles (uEVs), from 9 patients with cABMR compared to 33 patients with long-term graft survival (21). The authors found 46 proteins significantly differentially expressed in uEVs between the groups (p<0.001) (21). The list of studies used for these comparisons is summarized in **Supplementary Table S1A**, while the data files for each of these comparisons are included in **Supplementary Tables S2A–E**.

### Classical complement molecules are dominantly expressed in kidneys, blood and urine extracellular vesicles of patients with cABMR

**Figure 2:**
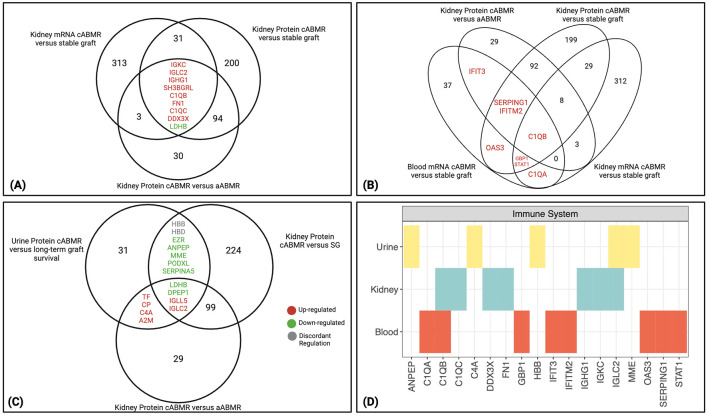
Molecules significantly altered in cABMR kidney and conserved in blood and urine. **(A)** Nine overlapping transcripts and proteins are identified between the 3 cABMR comparisons analyzing kidney tissue (19, 20). Eight were concordantly up-regulated between the 3 comparisons, while only LDHB was downregulated. **(B)** Eight overlapping transcripts and proteins were identified between 1 study analyzing blood in cABMR patients, and at least one of the 3 comparisons analyzing kidney tissue with cABMR (18–20). C1QB was shared between all comparisons. All molecules differentially expressed between cABMR and its comparator group were increased in cABMR. **(C)** Fifteen overlapping proteins between 1 study analyzing protein expression in urinary extracellular vesicles (EV) in cABMR and at least one of the 2 kidney tissue comparisons analyzing protein expression (20, 21). Seven proteins were downregulated in ABMR in at least two comparisons, 6 were upregulated, while 2 had discordant regulation between the studies (grey). **(A–C)** Upregulated proteins are shown in red font, while downregulated are in green. **(D)** From PathDIP analysis, the immune system pathway is enriched (FDR: BH<0.05) among molecules shared between biospecimens, particularly highlighting the classical complement system related molecules. aABMR, acute antibody-mediated rejection; cABMR, chronic antibody-mediated rejection; EV, extracellular vesicles.

Molecular expression was compared between the 4 cABMR studies analyzing kidney, urine, and blood. There were 9 genes/proteins shared between the 3 comparisons that analyzed kidney tissue, including immunoglobulins (IGKC, IGLC2, and IGHG1) and classical complement molecules (C1QB and C1QC) (**Figure 2A**). These molecules were increased in kidney tissue with cABMR as seen by the fold change values between comparisons highlighted in **Supplementary Table S3A**. Analysis of a prior scRNAseq dataset from living donor kidney biopsies revealed that C1QS genes are dominantly expressed within the mononuclear phagocyte cell clusters (22). A total of 137 genes/proteins inclusive of these 9 genes/proteins were shared between at least 2 of the 3 comparisons analyzing kidney tissue (**Supplementary Table S3A**, **Figure 2A**). Of these 137 molecules, 134 had concordant expression between comparisons, with 3 molecules (FTL, FTH1, and HSPA1B) up-regulated in cABMR in the scRNAseq dataset, but down-regulated at the protein level in Chauveau et al. for cABMR versus stable grafts (19, 20). The 137 molecules shared between the kidney tissue cABMR datasets were then used to identify enriched biological processes in gene ontology (GO) analysis, pathways (pathDIP), and microRNAs predicted to regulate these molecules (mirDIP), as outlined in the workflow of the cABMR analysis (**Supplementary Figure S1**) (11, 12, 23).

To address whether significantly differentially expressed molecules in cABMR were conserved across different biospecimens, a list of 45 genes differentially expressed between cABMR and stable grafts in blood was identified using the aforementioned studies (18). These 45 genes were compared to the full list of molecules from the 3 kidney tissue comparisons (**Supplementary Table S3B**). A total of 8 genes were shared and all were up-regulated in cABMR between at least 2 comparisons, while C1QB was the only molecule shared between all 4 comparisons (**Figure 2B**). Along with C1QB, other classical complement-related molecules (C1QA and SERPING1) were shared. The remaining molecules shared between the blood and at least one kidney comparison were all interferon-induced molecules (IFITM2, IFIT3, OAS3, GBP1, and STAT1) and expressed by the mononuclear phagocyte and endothelial cell clusters in the living donor kidney (22).

We next compared the 46 proteins found to be differentially expressed in uEVs between patients with cABMR and those with long-term graft survival (21) to the 2 kidney tissue protein analyses (20). Fifteen proteins were shared between at least 2 comparisons, with 4 proteins (LDHB, DPEP1, IGLL5, and IGLC2) shared between all 3 comparisons (**Figure 2C**, **Supplementary Table S3C**). Finally, 13 of 15 proteins shared between the urine analysis and at least one of the kidney protein analyses had concordant direction of expression (HBB and HBD were discordant). Structural proteins, such as podocalyxin (PODXL) and ezrin (EZR), were down-regulated in cABMR in both the uEVs and the kidney tissue. These proteins are involved in cell adhesion and structural integrity of cells and tissue (24, 25). PODXL and EZR were highly expressed in podocyte cell clusters in living donor kidneys, and PODXL was also expressed in endothelial cell clusters (22, 26). PODXL is glycosylated and sulfated to produce a negatively charged structure essential for maintaining the filtration barrier (24). EZR is a part of the ezrin/radixin/moesin (ERM) family of proteins, acting as an intermediary between the actin cytoskeleton and plasma membrane to support the podocyte structure near the glomerular filtration barrier (25). The significant down-regulation (**Supplementary Table S3C**) of these proteins therefore underlines loss of important structural components of the glomerular filtration barrier in cABMR.

From the molecules found to be shared between these comparisons and biospecimens, GO and pathDIP searches were done to inform which processes and pathways were enriched (FDR: BH <0.05) (12, 23). The 137 genes/proteins found to be shared between at least 2 of 3 kidney tissue comparisons had 109 enriched biological processes (q<0.05) (**Supplementary Table S4A**). Using pathDIP, 318 pathways were found to be significantly enriched within the kidney (q<0.05) (**Supplementary Table S4B**). Forty-two biological processes (**Supplementary Table S4C**) and 40 pathways (**Supplementary Table S4D**) were enriched among the 45 genes from the PBMC analysis. Finally, 54 biological processes (**Supplementary Table S4E**) and 110 pathways (**Supplementary Table S4F**) were enriched among the 46 proteins from the uEV analysis.

Comparison of the GO IDs and pathways shared between all 3 biospecimens resulted in 7 biological processes (**Supplementary Figure S2**) and 14 pathways (**Supplementary Figure S3**). The most consistent observations from these GO terms and pathways highlight the immune system molecules, particularly the classical complement system. **Figure 2D** highlights the immune system pathway found to be significantly enriched in PathDIP with complement related molecules shared across bio-specimens, including C1Q molecules, C4A, immunoglobulins, and SERPING1 (kidney: q=1.42E-06, blood: q=1.83E-08, urine: q=3.44E-05).

To computationally predict regulators of our key molecules overexpressed in cABMR, we focused on microRNAs. Using mirDIP (11), we identified microRNAs predicted with ‘high’ confidence to regulate the 137 molecules in cABMR kidney tissue, 45 genes in cABMR PBMCs, and the 46 uEV proteins significantly differentially expressed in cABMR (**Supplementary Tables S5A-C**). We then leveraged NephroDIP to identify studies that specifically evaluated microRNAs in cABMR. The PBMC microarray analysis that was used to inform the blood signature found 16 microRNAs down-regulated in cABMR compared to control patient PBMCs (18) (**Supplementary Table S2F**). Another comparison analyzed serum microRNA expression in patients with cABMR compared to patients with normal allograft function and found 19 differentially expressed microRNAs (27) (**Supplementary Table S2G**). Finally, a microRNA study by Franzin et al. analyzed the microRNA expression between aABMR and cABMR in blood EVs and found 9 differentially expressed microRNAs (28) (**Supplementary Table S2H**). These microRNA studies are listed and summarized within **Supplementary Table S1A**. The great majority of microRNAs were down-regulated in cABMR, with the exception of the analysis from Franzin et al. that had mostly up-regulated microRNAs in cABMR.

**Figure 3:**
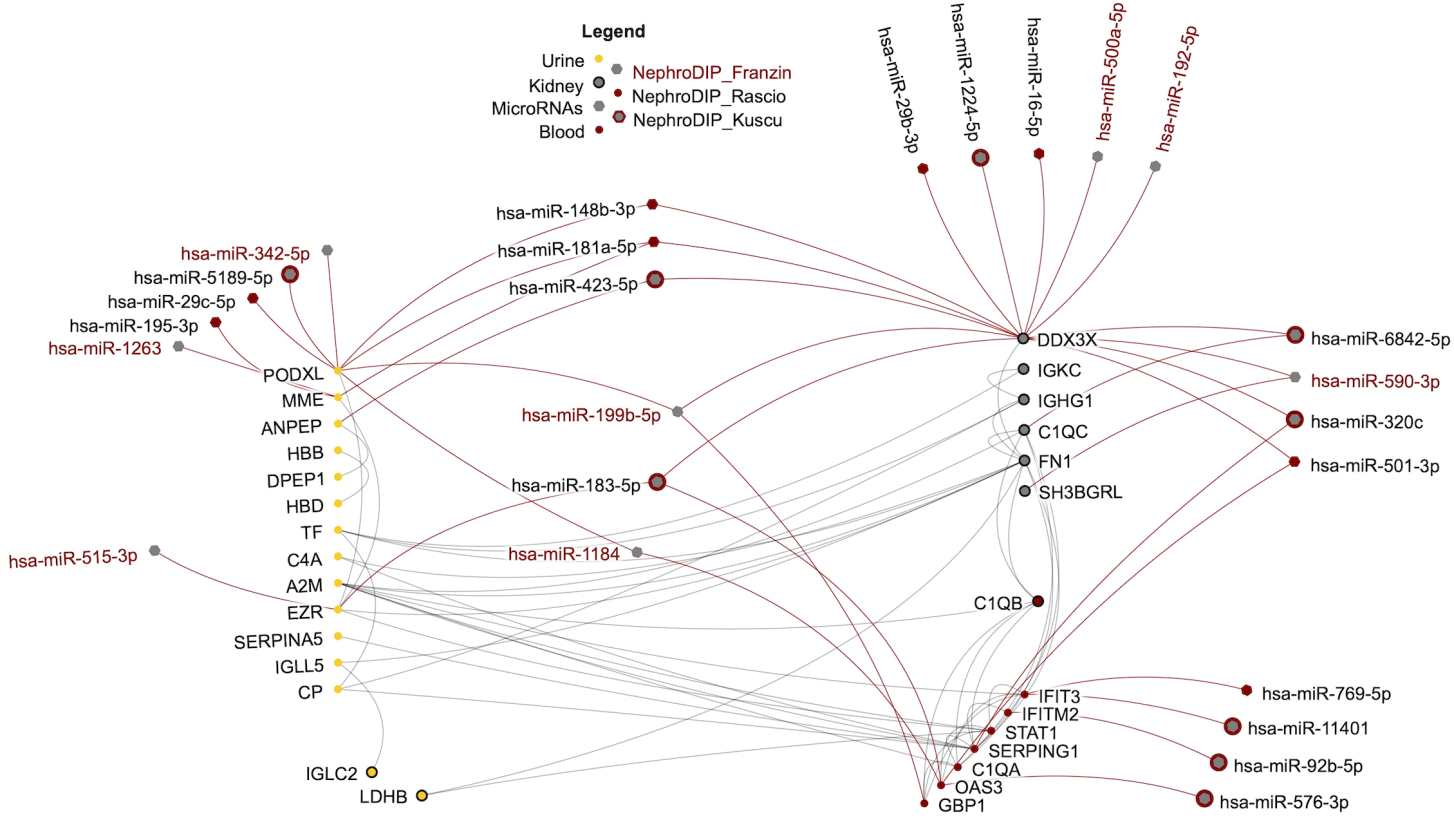
Network of shared transcripts, proteins, and microRNAs differentially expressed in cABMR and predicted to regulate the shared molecules. The protein-protein interactions between the molecules are shown in grey and the regulation by microRNAs is in red. Each node in the network represents a molecule found in NephroDIP, while the edges represent either direct interaction between molecular nodes, or evidence of regulation (e.g. microRNA and gene). Proteins identified in urine are shown as yellow nodes. Red nodes indicate molecules identified in blood.

To visually highlight this cABMR signature across biospecimens, a network was produced using NAViGaTOR ver. 3.0.19 (13). This included the 9 molecules shared between all 3 kidney tissue analyses, the 8 molecules shared between blood and kidney, the 15 proteins shared between the urine and kidney, and the 25 microRNAs found to overlap between the 3 NephroDIP studies and mirDIP predictions (**Figure 3**). The classical complement system dominates the network. Furthermore, 9 microRNAs connect to the up-regulated interferon related molecules (OAS3, IFITM2, IFIT3, SERPING1, GBP1) and 12 microRNAs connect to the down-regulated proteins between the uEVs and kidney glomerular tissue (PODXL, MME, ANPEP, and EZR). Six microRNAs were predicted to regulate PODXL. Of these 6 microRNAs, hsa-miR-199b-5p and hsa-miR-342-5p were significantly up-regulated in cABMR compared to aABMR patients. This pattern of expression may suggest inhibitory effect of the microRNAs, resulting in decreased PODXL expression in cABMR (28, 29).

### NephroDIP reveals LGALS1 interactome signatures in the settings of fibrosis and immunosuppression

LGALS1 is an immunomodulatory protein that we previously found to be increased in the glomeruli of aABMR biopsies compared to other forms of kidney injury (30). Additionally, we demonstrated that universal anti-HLA class I antibodies increased transcriptional expression and protein secretion of LGALS1 in kidney glomerular microvascular endothelial cells *in vitro*. Other studies have shown the ability of LGALS1 to induce apoptosis in T-cells and prevent T-cell migration across the tumor endothelium (31, 32), while the addition of recombinant LGALS1 prolonged the survival of the kidney allografts and was required to induce tolerance in B-cells, in preclinical models (33, 34).

Given the potential role of LGALS1 in kidney transplant rejection, we used NephroDIP to characterize the expression of LGALS1 as well as its interactors and regulators across published studies with high-throughput data. To identify LGALS1 interactors, the Integrated Interactions Database (https://ophid.utoronto.ca/iid), IID version 2021-05 (35), was searched for experimentally validated interactors of LGALS1 in the human kidney (**Supplementary Table S6**). This list was then searched in the Catrin version 2 database (https://ophid.utoronto.ca/catrin) to generate a list of enriched transcription factors predicted to regulate LGALS1 and its interactors (**Supplementary Table S7**). NephroDIP was then searched using the gene identifier, LGALS1, and the UniprotKB ID, P09382, which resulted in 16 studies with 29 comparisons (**Supplementary Figure S4**). Studies in NephroDIP that contained LGALS1 expression mainly investigated interstitial fibrosis and tubular atrophy (IFTA) (2 studies) or immunosuppression (3 studies). The list of studies used for these comparisons can be found in **Supplementary Table S1B**. Downloaded datasets from these 5 studies were queried for LGALS1 and its interactors and transcriptional regulators. LGALS1 interactors and regulators whose expression changed in the same direction as LGALS1 and were conserved between datasets, were then analyzed using pathDIP to assess the enriched pathways in the setting of IFTA or immunosuppression. This workflow for each use case is visualized in **Supplementary Figure S4**.

### Expression of LGALS1 and its key interactors and regulators is increased in kidney allograft fibrosis

**Figure 4:**
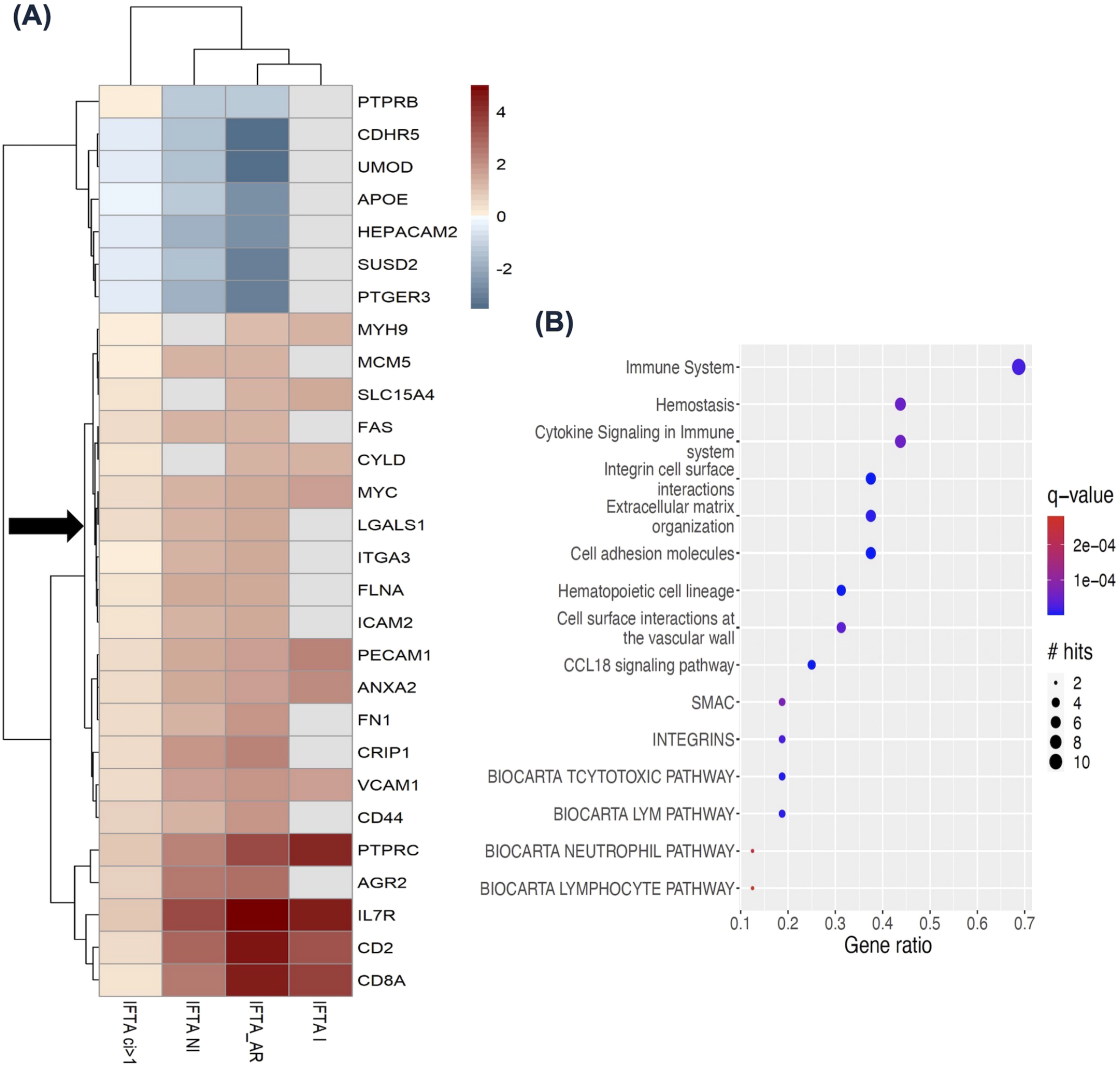
LGALS1 and its interactome expression in the kidney allografts with interstitial fibrosis and tubular atrophy (IFTA), from Venner et al., 2016 and Modena et al., 2016 (36, 37). **(A)** Heatmap showing 28 LGALS1 interactor genes differentially expressed in at least 3/4 comparisons to normally functioning kidney transplants or kidney transplants with mild/no IFTA (ci</=1). Red color indicates increased expression and blue color indicates decreased expression. LGALS1 is denoted by black arrow. **(B)** Top 15 pathways enriched (FDR: BH<0.01) among the concordant interactors from **(A)** (red). The size of the circle indicates the number of LGALS1 interactor**s** in the pathway, and the color indicates significance. IFTA, interstitial fibrosis and tubular atrophy; IFTA ci>1, interstitial fibrosis and tubular atrophy with at least moderate interstitial fibrosis score; IFTA-I, interstitial fibrosis and tubular atrophy with inflammation; IFTA-NI, interstitial fibrosis and tubular atrophy with no inflammation; IFTA-AR, interstitial fibrosis and tubular atrophy with acute rejection.

We first examined studies with IFTA to answer the question: *What happens to the expression of LGALS1 and its interactors in the kidney allograft in the setting of fibrosis, and which transcription factors may regulate LGALS1 in kidney fibrosis?* The IFTA search yielded 2 kidney studies with 4 comparisons. The first study compared kidney transplant indication biopsies with at least moderate IFTA (ci>1) (n=200) to biopsies with mild/no IFTA (ci</=1) (n=199) (36). We included ‘late’ biopsies after 1-year post-transplant, because this time post-transplant coincided with the 3 comparisons of IFTA from the second study. Late biopsies with ci>1 were compared to biopsies with ci</=1 using bulk mRNA expression in the kidney, resulting in 12,403 differentially expressed genes (DEGs) found (p<0.05) between the groups (**Supplementary Tables S8A**). The second study contained 3 comparisons: IFTA with acute rejection (n=29), IFTA with inflammation (n=10), and IFTA without inflammation (n=42) compared to normally functioning transplants (n=99) (37). This study found 6,229 DEGs in IFTA with acute rejection, 1,424 DEGs in IFTA with inflammation, and 3,280 DEGs in IFTA without inflammation, all compared to normally functioning transplants (p<0.05) (**Supplementary Tables S8B–D**). The DEGs from both studies were then interrogated for known LGALS1 interactors (**Supplementary Table S6**) and transcriptional regulators (**Supplementary Table S7**). Twenty-eight LGALS1 interactors, including LGALS1, are shown with their gene expression fold change in **Figure 4A**, while 46 transcription factors targeting LGALS1 and its interactors are shown in **Supplementary Figure S5A**. These LGALS1 interactors and transcription factors were differentially expressed in at least 3/4 comparisons. Twenty-one out of 28 LGALS1 interactors were significantly up-regulated in IFTA, indicated by red color in the heatmap (**Figure 4A**). To determine the likely cellular origin of these upregulated LGALS1 interactors, we compared them to the prior scRNAseq dataset from living donor kidney biopsies (22). Genes such as *PECAM1, VCAM1, ICAM2, FN1, CD44, CD2, CD8A*, and *IL7R* were expressed in endothelial and immune cells (CD8^+^ and CD4^+^ T-cells, natural killer (NK) cells and mononuclear phagocytes) (22), suggesting that these cells may be the main actors producing the LGALS1 interactome and that the expression of these genes is altered in the setting of fibrosis. Genes found to have significant differential expression in IFTA across the above comparisons, as well as concordant expression with LGALS1, were subsequently searched in pathDIP to identify enriched pathways (12). The 21 interactor genes concordant to LGALS1 yielded 64 enriched pathways (hypergeometric test, FDR: BH <0.01) (**Supplementary Table S9A**). Several important pathways for tissue remodeling in fibrosis (38), such as cytokine signalling (q=5.48E-05), extracellular matrix organization (q=8.19E-06), cell surface interaction (q=2.97E-08) and focal adhesion (q=3.36E-07) are seen in the graph of the top 15 pathways enriched among LGALS1 interactors (**Figure 4B**). Similarly, 41/46 transcription factors were up-regulated in IFTA and displayed the same direction of change as LGALS1, denoted by their red color on the heatmap (**Supplementary Figure S5A**). Interestingly, several of these transcription factors are direct regulators of the concordant interactor genes above, such as STAT1, STAT3, RUNX3, HIF1A, and MYC. These transcription factors have also been implicated in signaling mechanisms related to fibrosis (39–42). In addition, the 41 transcription factor genes concordant to LGALS1 expression yielded a total of 259 enriched pathways in pathDIP (hypergeometric test, FDR: BH <0.01) (**Supplementary Table S9B**). Among the top 15 enriched pathways were epithelial–mesenchymal transition regulators (q=4.35E-07) and transforming growth factor beta (TGF-β) signaling (q=6.44E-08) (**Supplementary Figure S5B**), known to be important in fibrosis (43). Overall, our findings suggest that the protein network anchored around LGALS1 is increased in kidney allograft fibrosis and is predominantly expressed in endothelial and immune cells such as mononuclear phagocytes, CD8^+^ T-cells, and NK cells, hinting at their potential involvement in fibrosis.

### The LGALS1 interactome is increased in innate immune cells and decreased in T-cells in the blood during immunosuppression post-transplantation

**Figure 5:**
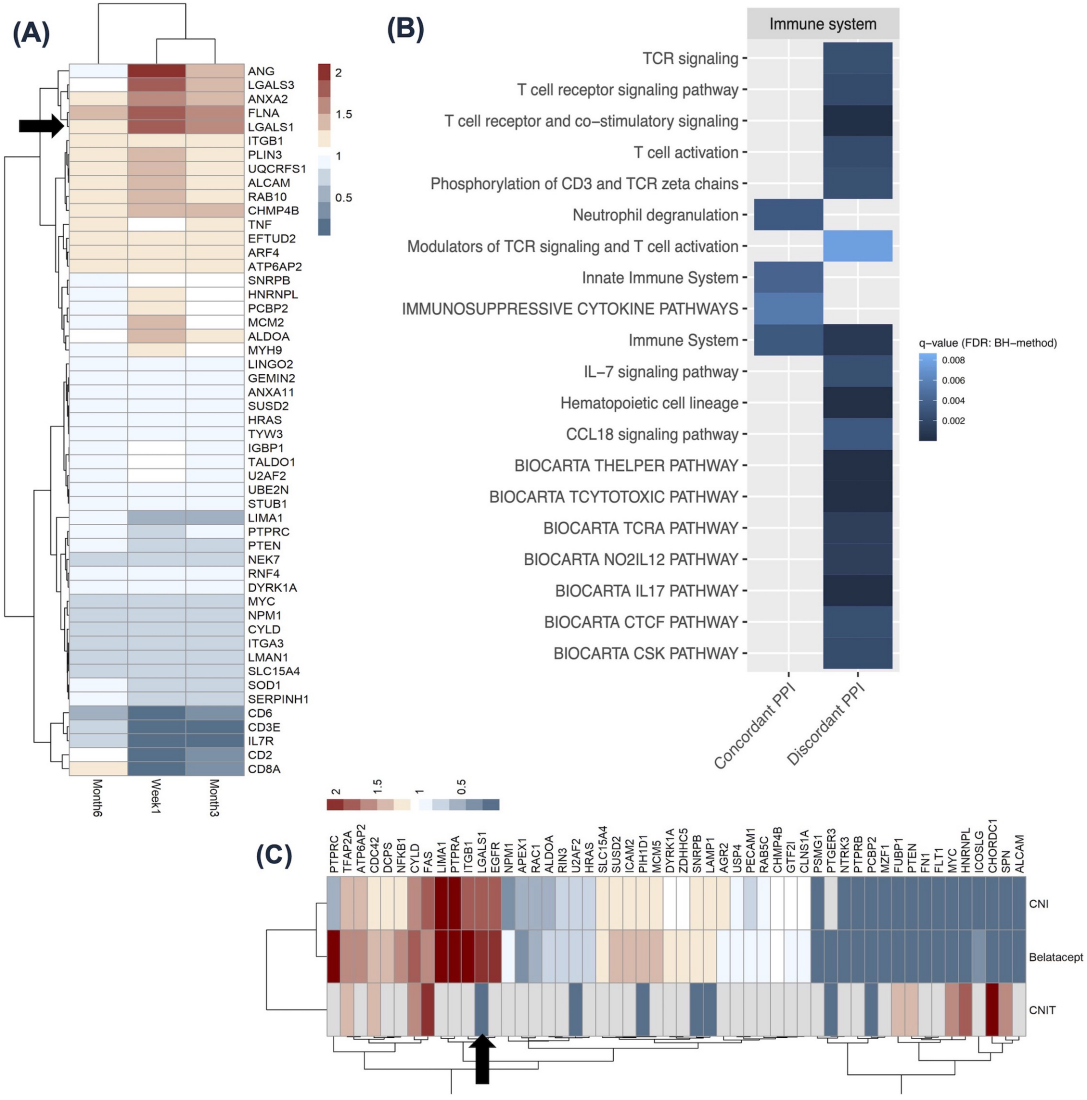
LGALS1 and its interactome expression in the context of immunosuppression. **(A)** Heatmap showing 51 LGALS1 interactor genes significantly differentially expressed in at least 2 time points in PBMCs from patients on standard post-transplant immunosuppression, from Dorr et al., 2015 (44). Red indicates high expression and blue indicates low expression, relative to pre-transplantation values. LGALS1 is denoted by black arrow. The time after transplant when PBMC transcriptome was analyzed is shown for each column in the heatmap. **(B)** Heatmap showing 20 enriched immune system pathways for concordant and discordant LGALS1 interactors from **(A)** (FDR: BH <0.01)​. Darker blue indicates lower q-value. **(C)** Heatmap shows 53 LGALS1 interactor genes significantly differentially expressed in the kidney allograft, in at least 2 comparisons in patients immunosuppressed with either CNI, Belatacept, or patients demonstrating CNI toxicity, from Vitalone et al., 2014 and Rhone et al., 2021 (45, 46). Red indicates high expression and blue indicates low expression, relative to pre-implantation biopsies or stable graft biopsies. PBMC, peripheral blood mononuclear cell; PPI, protein-protein interactor; CNI, calcineurin inhibitor; CNIT, calcineurin inhibitor toxicity.

We next utilized NephroDIP to answer the following question: *How does immunosuppression post-transplantation affect LGALS1 expression and its interactors in the blood?* Dorr et al. examined the effect of immunosuppression on the PBMC transcriptome in 32 adult living donor kidney transplant recipients without previous rejection or evidence of rejection at the time of sampling, treated with anti-thymoglobulin induction, calcineurin inhibitor (CNI), and mycophenolate mofetil with or without steroids (44). PBMC gene expression was evaluated at 1 week, 3 months, and 6 months post-transplant compared to pre-transplant levels (**Supplementary Tables S10A–C**). We evaluated whether LGALS1 and its interactors were significantly altered in PBMCs, at any of the time points compared to pre-transplant. Fifty-one LGALS1 interactor genes (**Supplementary Table S6**) were significantly differentially expressed in at least 2 comparisons. LGALS1 was significantly up-regulated following immunosuppression at all time-points (denoted by black arrow) (**Figure 5A**), and 14/51 interactors showed similar expression to LGALS1, with a fold change>1 in at least 2 time-points, denoted by the red color. The fold changes for this group of genes were the highest at 1-week post-transplant and diminished with time. We utilized a scRNAseq living donor kidney dataset to assess the cells most likely expressing these genes (22). Genes such as *LGALS3, RAB10, ITGB1*, and *FLNA*, exhibited the highest expression in innate immune clusters such as the mononuclear phagocytes and NK cells. Interestingly, 5/51 interactors, seen in the heatmap in **Figure 5A**, showed a discordant expression to LGALS1, with fold change<0.5 in at least 2 time-points, indicated by the dark blue color. These 5 LGALS1 interactor genes exhibited the most decreased expression during immunosuppression and appeared to be specific to T-cell (particularly CD8+) clusters. This suggests that LGALS1 interactome in PBMCs appears to reflect the degree of immunosuppression in T-cells, whereas the opposite pattern of expression is notable in innate immune cells.

We next evaluated pathways enriched among genes concordant or discordant with LGALS1 (12). The 51 interactors differentially expressed in at least 2 time points had 15 enriched pathways when searching by concordant genes and 30 pathways when searching by discordant genes (FDR: BH <0.01) (**Supplementary Tables S11A, B**). The tile plot in **Figure 5B** highlights LGALS1 interactors with concordant expression to LGALS1 (left side) participate in innate immune system pathways, while those with discordant expression to LGALS1 (right side) participate in humoral immunity, and T-cell receptor signaling and activation (**Figure 5B**). This further supports our analysis of LGALS1 interactome in the scRNAseq dataset and suggests that this group of genes may reflect the known effects of current immunosuppression on T-cells, while intriguingly highlighting the potential lack of direct effect on innate immune cells.

### CNI or Belatacept immunosuppression results in differential expression of the LGALS1 interactome in post- versus pre-transplant kidney allograft biopsies

We next used NephroDIP to determine whether the type of immunosuppression post-transplantation affects LGALS1 expression, its interactors, and its regulators in the kidney. Two studies containing 3 comparisons in the context of immunosuppression were identified in NephroDIP. The first study, by Vitalone et al., examined gene expression in routine surveillance kidney biopsies at implantation or 12 months after transplantation collected for the BENEFIT and BENEFIT-EXT study (45). The study compared 17 patients receiving CNI and 18 patients receiving Belatacept to 26 pre-implantation control biopsies. IFTA scoring for the 12-month biopsies revealed that 13% of the 15 reported biopsies in the Belatacept cohort and 35% of the 17 biopsies in the CNI cohort had a ci score >/= 1 (45). We were interested in this since the previous case showed an increase in LGALS1 expression for ci scores >1, however this observed difference in fibrosis between the 2 cohorts did not cause a large difference in expression of the investigated genes (**Supplementary Tables S12A, B**) (45). The second study, by Rhone et al., examined gene expression in formalin-fixed paraffin-embedded pediatric kidney biopsies of 11 patients with CNI toxicity (CNIT) compared to 12 stable graft biopsies (**Supplementary Table S12C**) (46). IFTA scoring for this CNIT cohort was not provided in the study. However, in this study, CNIT was defined as histological features of isometric vacuolization of the proximal convoluted tubules, nodular hyalinization of arterioles and small arteries, or striped interstitial fibrosis, all in the absence of IFTA or rejection (46). Fifty-three interactor genes (**Supplementary Table S6**) and 131 transcription factors (**Supplementary Table S7**) were differentially expressed in at least 2/3 comparisons from 2 studies above (p<0.05). The heatmap in **Figure 5C** highlights significantly increased LGALS1 expression in the CNI and Belatacept groups, but significantly decreased LGALS1 expression in the CNIT group, denoted by the black arrow. **Figure 5C** also illustrates 19 interactor genes with similar expression to LGALS1 in CNI and Belatacept groups, denoted by their red color. Conversely, 25 interactor genes showed the opposite expression to LGALS1 in CNI and Belatacept, denoted by their blue color. Notably, only a subset of genes was detected in CNIT. Moreover, 45 transcription factors (**Supplementary Figure S6A**) displayed the same direction of expression as LGALS1 in CNI and Belatacept (shown in red), while 56 transcription factors had the opposite expression to LGALS1 in the 2 groups (shown in blue). Only 23/131 transcription factors targeting LGALS1 and its interactors were identified in the CNIT group, and the direction of expression tended to be distinct in this group (**Supplementary Figure S6A**). Overall, kidneys exposed to CNI and Belatacept had more similar transcriptional profiles of LGALS1 interactors and transcription factors to each other than to kidneys with CNIT.

We next examined the enriched pathways among the concordant and discordant LGALS1 interactors. LGALS1 interactor genes concordant in expression to LGALS1 had 56 enriched pathways, and those discordant to LGALS1 expression had 127 enriched pathways (FDR: BH <0.01) (**Supplementary Table S13**). The top 15 enriched pathways of these concordant interactors included CKAP4 signaling pathway (q=5.97E-05), TROP2 regulatory signaling pathway (q=3.91E-05), RAC1/PAK1/p38/MMP2 pathway (q=9.84E-05), and death receptor pathways (q=3.00E-03) (**Supplementary Figure S6B**). Discordant interactor genes included PI3K-Akt pathway (q=2.76E-04), TROP2 regulatory signaling pathway (q=6.50E-07), RAC1/PAK1/p38/MMP2 pathway (q=2.26E-06), and regulated cell death (RCD) genes (q=1.04E-04) (**Supplementary Figure S6C**). Interestingly, CKAP4 ligation can initiate the signalling cascade of the PI3K-Akt pathway (47), which is the top pathway enriched for discordant interactors of LGALS1. The concordant and discordant interactor pathways also shared death-related genes, TROP2 signalling, and RAC1/PAK1/p38/MMP2 pathways among their top 5 enriched pathways. These findings suggest that the LGALS1 interactome may be involved in the critical cell death and signalling pathways activated during immunosuppression in the kidney.

## Discussion

Here we describe the creation of NephroDIP v1.0, a data integration portal for human kidney transplantation research that includes metadata and molecular expression from 134 studies. As we have demonstrated, all queries in NephroDIP can be directly connected to external tools, including Drugst.One, NAViGaTOR, mirDIP, pathDIP, IID, and Catrin (11–14, 35), for a more comprehensive insight on interactions or regulation.

We first used NephroDIP to define the key conserved molecular signatures of cABMR, the most important cause of premature kidney allograft loss (15, 48). The upregulation of early stage complement molecules in cABMR was conserved across studies, and evident from enriched biological processes and pathways. This finding was interesting, considering that many patients with cABMR lack C4d deposition, which is no longer required for cABMR diagnosis (16). Furthermore, the presence of C1Q molecules within mononuclear phagocytes (22) may suggest that these cells locally produce C1Q within the kidney (49, 50), though this speculation would have to be formally tested. The overlap of interferon-induced molecules between the blood and kidney further supports the pivotal role of interferon signalling in cABMR, which increases inflammation, recruits immune cells, enhances HLA expression, and increases vascular permeability in ABMR (51–53). Furthermore, as 7/9 microRNAs targeting these molecules were down-regulated, this suggests decreased inhibitory action on the interferon signatures (29) as a potential novel regulatory mechanism of interferons. Additionally, the 13/15 concordantly expressed proteins between the uEVs and glomerular tissue highlight the potential of uEVs to reflect a glomerular signature. As others suggest, uEVs could serve as a less invasive alternative to biopsies in diagnosing ABMR; however, further insight is needed to understand their origin and function (54, 55). Of the concordant proteins, the decrease in structural proteins, such as PODXL and EZR, may signify the loss of, or structural injury to podocytes in cABMR, supported by previous studies (30, 56). Finally, in addition to decreased PODXL expression in glomeruli and uEVs, several microRNAs that regulate PODXL were increased in cABMR. One of them, hsa-miR-199b-5p, was implicated in epithelial-mesenchymal transition, a process PODXL has been previously linked to (24, 57–61). Similarly, hsa-miR-342-5p, was implicated in endothelial-mesenchymal transition, which was previously recognized in kidney ABMR pathogenesis (62, 63). Of note, PODXL is expressed in both endothelial cells and podocytes (22). In summary, we have identified several conserved molecular features associated with cABMR that deserve further mechanistic studies.

We next queried NephroDIP to examine the expression of LGALS1 related signatures in kidney IFTA. The expression of LGALS1 and most of its interactors and transcription factors were increased in IFTA. LGALS1 has been previously linked to fibrosis and is important for repair of damaged tissues (64). Tissue repair is a complex process, and fibroblasts play a major role, with their ability to produce collagen and extracellular matrix components necessary for cell adhesion and wound closure (65). However, overactivation of fibroblasts and their conversion to myofibroblasts can result in deposition of excess collagen and extracellular matrix, turning wound healing into fibrosis. Although the contribution of endothelial-mesenchymal transformation to fibrosis in the kidney is controversial, the endothelial cell signatures seen in our analysis could conceivably serve as a source of fibroblasts (66). Moreover, the increased LGALS1 seen in IFTA could itself contribute to further activation of existing fibroblasts and thereby worsen fibrosis. Several studies in the lung have shown that inhibition of LGALS1 can decrease fibrotic injury through apoptosis and decreased accumulation of fibroblasts (67, 68). LGALS1 inhibition post-transplantation could potentially represent a therapeutic target for decreasing IFTA in the kidney, although its role in kidney allograft fibrosis will first have to be validated.

Lastly, we examined the expression of LGALS1 and its interactome in the context of immunosuppression. An interesting pattern of LGALS1 interactome expression emerged, whereby molecules increased in immunosuppression were predicted to be expressed by innate immune cells, while those decreased in immunosuppression were predicted to be expressed by adaptive immune cells. These findings are consistent with the known mechanism of action of current immunosuppressants, which predominantly target T-cells, and may reflect the overall decrease in T lymphocytes (69–71). LGALS1 is known to be expressed by antigen presenting cells and plays an important role in immune tolerance, pro-resolving immune response, and killing of activated T-cells (31, 72–74). Given the ability of LGALS1 to interact with T-cells, the concurrent disruption of LGALS1 and related T-cell signalling molecules in immunosuppression may reflect a previously unrecognized mechanism of immunosuppression through LGALS1 directly, a hypothesis that warrants further study. Finally, we found a stark difference in gene expression of LGALS1 and its interactors in the kidney in patients treated with CNI or Belatacept, compared to kidneys exhibiting CNIT. Given the distinct mechanism of action for each drug, it is even more striking that Belatacept and CNI had a more similar effect on the LGALS1 interactome to each other than when compared to CNIT, though the difference could be attributed to adult vs. pediatric patients studied and other potential confounders. Both forms of immunosuppression were shown to influence pathways enriched among LGALS1 interactors in the kidney. Concordant and discordant interactors were enriched for shared pathways, including CKAP4/PI3K-Akt, TROP2, RAC1/PAK1/p38/MMP2, and cell death pathways. Interestingly, LGALS1 can both activate and inhibit the PI3K-Akt pathway, in a context-dependant manner, which plays a role in migration and proliferation (75, 76). Furthermore, it is known that TROP2 and RAC/p38 can also participate in the PI3K-Akt pathway (77, 78). These findings support the participation of the LGALS1 interactome in cell death and signalling pathways present during immunosuppression in the kidney. Future studies may investigate the role of LGALS1 in CKAP4 and TROP2 signalling and PI3K-Akt pathway activation during kidney immunosuppression, as well as investigate if LGALS1 manipulation in specific immune cells increases the effectiveness of immunosuppressive therapy.

The work presented here has a number of strengths. NephroDIP is a unique resource for future studies, as it is open-access and contains curated, annotated and standardized high-throughput data on the kidney allograft, within one portal. This enables easy comparisons of molecular signatures across studies, and integration of multi-omics data. Additionally, seamless integration with other databases and portals enhances the analysis of signatures and hypothesis generation.

Our work also has a few limitations. NephroDIP is limited by the inherent heterogeneity among studies. The data imported are only as good as the original studies themselves. Additionally, study curation resulted in <4% of studies being included across each molecular search, revealing a small number of studies that were deemed high quality based on the inclusion criteria. A major limitation present within the literature, emphasized by this curation, is the lack of available data. A total of 320 studies that met all inclusion criteria could not be added to NephroDIP because they did not include the data analyzed within their publication. Adding these studies would have increased the information available within NephroDIP by almost 3-fold, underscoring the need to promote sharing and transparency of data within the scientific community. Expanded data resources is not the only benefit; considering the alarming rate of retractions (79), and if the publication does not include data, the correctness of the findings cannot be verified.

In conclusion, NephroDIP v1.0 is an open access platform that will be a beneficial resource to users in the field of kidney transplantation, particularly because of the expansion of high-throughput “omics” research in recent years. NephroDIP enables fast and specific searches of kidney transplant literature from Jan 2002 to May 2023, due to its standardized annotation built from many diverse datasets across the literature. NephroDIP will continue to receive updates to the published literature, with users allowed to submit manuscripts that fit the inclusion criteria.

## Data Availability

The datasets presented in this study can be found in online repositories. The names of the repository/repositories and accession number(s) can be found in the article/**Supplementary Material**.
