# Supplementary material for: Molecular landscape of kidney allograft tissues data integration portal (NephroDIP): a curated database to improve integration of high-throughput kidney transplant datasets
Source: Front Immunol. 2024 Sep 27;15:1469500. doi: 10.3389/fimmu.2024.1469500 (PMC11466753; doi:10.3389/fimmu.2024.1469500)
Supplement: Supplementary file 1 [file DataSheet1.pdf]

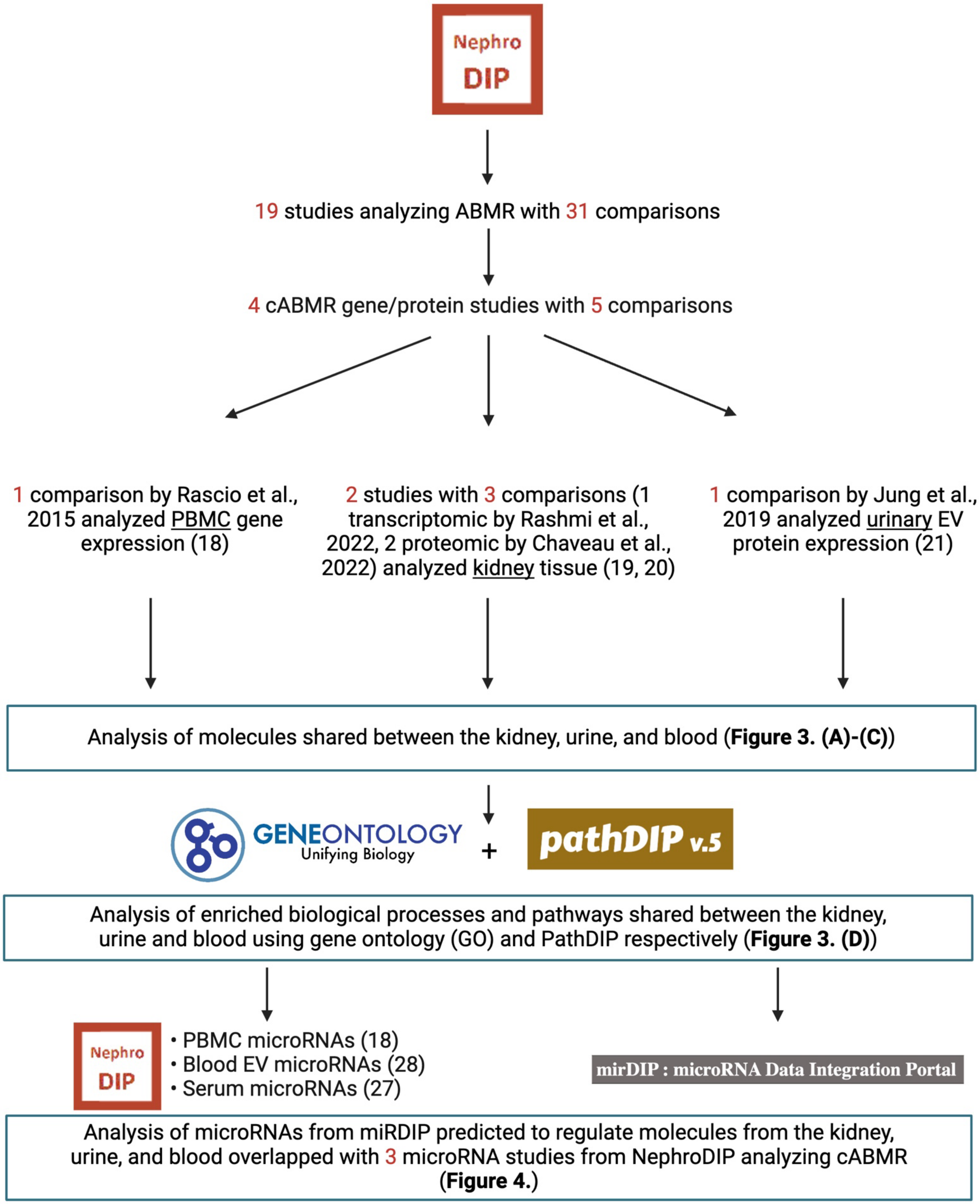

Supplementary Figure 1. The workflow leading to identification of the conserved molecular signatures in chronic antibody-mediated rejection (cABMR) across studies. ABMR, antibody-mediated rejection; cABMR, chronic ABMR; GO, Gene Ontology; PBMC, peripheral blood mononuclear cells; EV, extracellular vesicles.

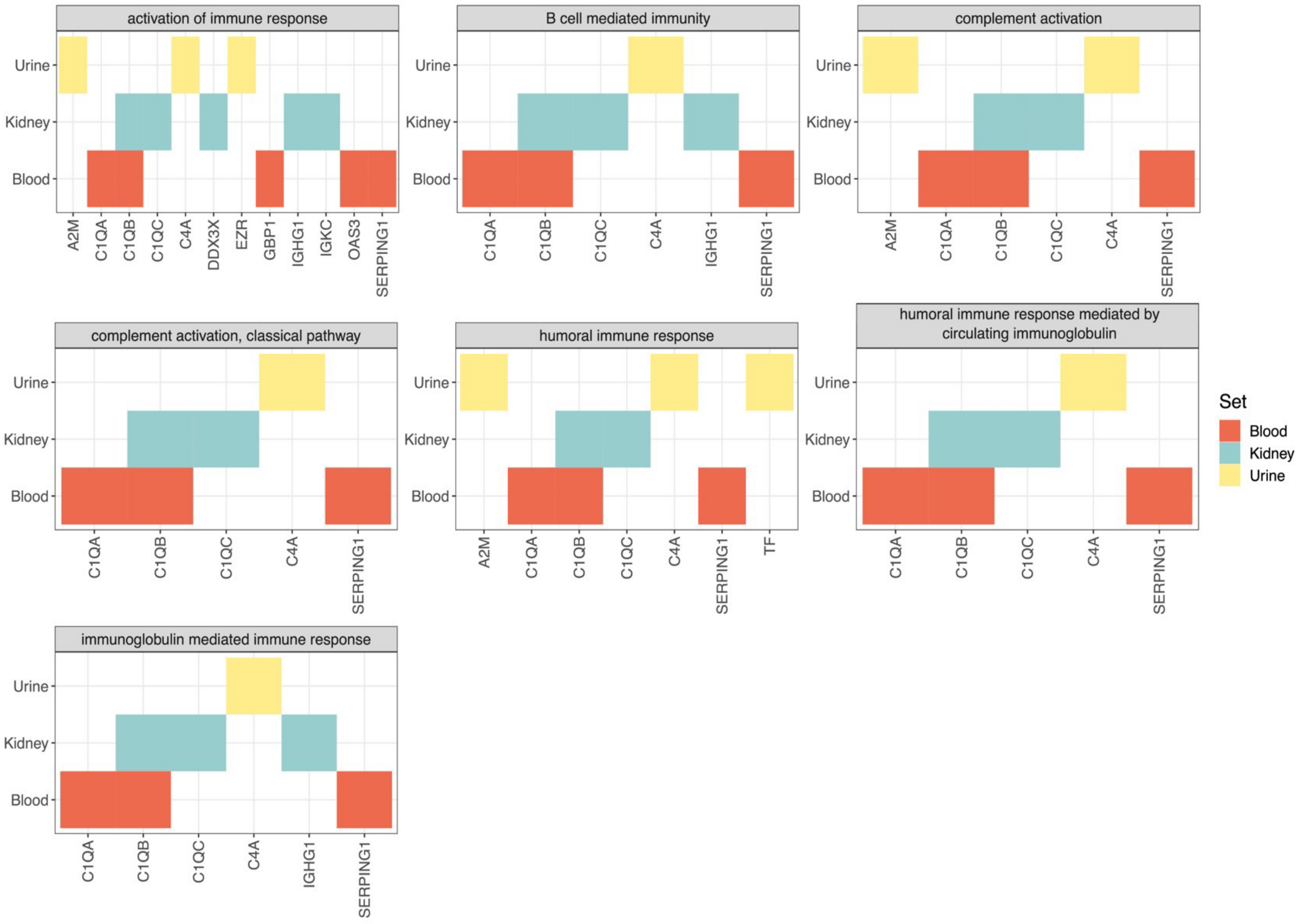

Supplementary Figure 2. Seven biological processes (FDH:BH<0.05) overlapping between the Gene Ontology searches of the 137 kidney molecules significantly altered in cABMR, 45 genes significantly altered in cABMR in the peripheral blood mononuclear cell analysis, and 46 proteins significantly differentially expressed in the urinary extracellular vesicles from patients with cABMR. The column highlights the genes differentially expressed and present in the biological process, while the row highlights the biospecimen. Red indicates genes found in the blood, blue indicates genes found in the kidney tissue, and yellow indicates genes found in the urine. cABMR, chronic antibody mediated rejection.

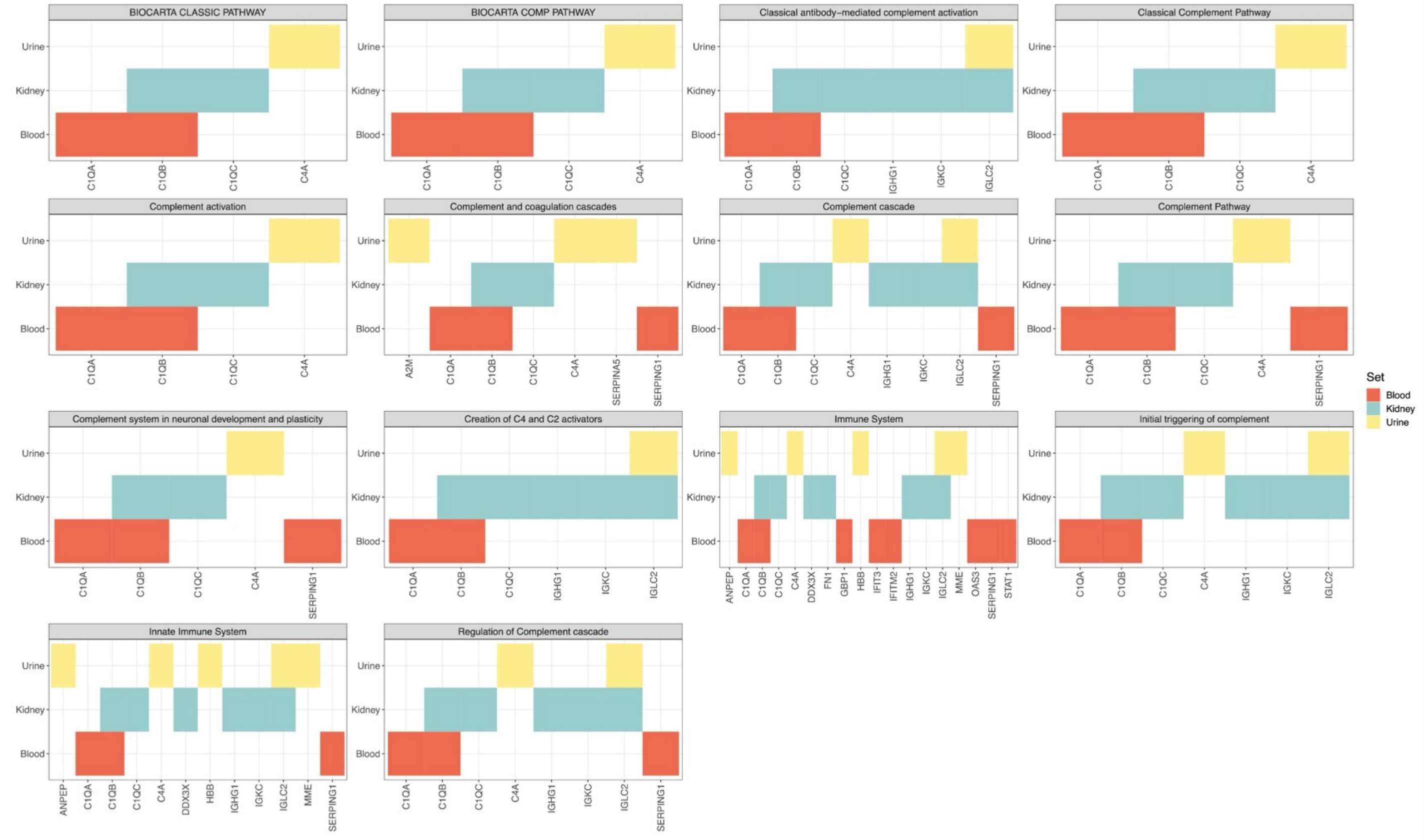

Supplementary Figure 3. Fourteen pathways (FDH:BH<0.05) overlapping between the PathDIP searches of the 137 kidney molecules significantly altered in cABMR, 45 genes significantly altered in cABMR in the peripheral blood mononuclear cell analysis, and 46 proteins significantly differentially expressed in the urinary extracellular vesicles from patients with cABMR. The column highlights the genes differentially expressed and present in the biological process, while the row highlights the biospecimen. Red indicates genes found in the blood, blue indicates genes found in the kidney tissue, and yellow indicates genes found in the urine. cABMR, chronic antibody mediated rejection.

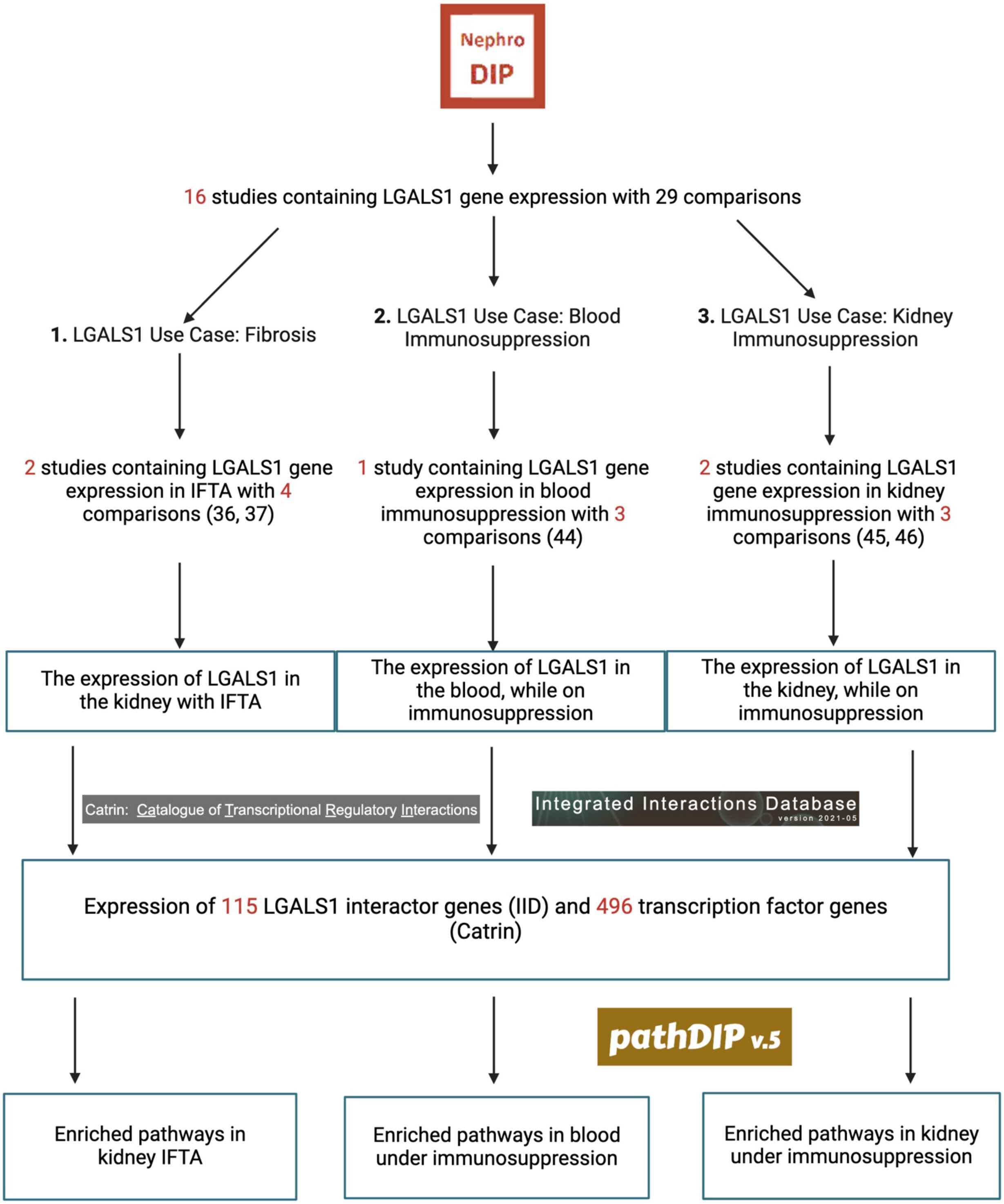

Supplementary Figure 4. The workflow to evaluate LGALS1 in kidney transplantation: 1. LGALS1 in kidney allografts with interstitial fibrosis and tubular atrophy (IFTA) integrating data from Venner et al., 2016 and Modena et al., 2016 (36, 37), 2. LGALS1 in blood PBMCs in the context of immunosuppression from Dorr et al., 2015 (44), 3. LGALS1 in kidney allograft exposed to different immunosuppression regimens: CNI treatment or Belatacept, or CNI toxicity integrating data from Vitalone et al., 2014 and Rhone et al., 2021 (45, 46). Use cases were generated by separating studies and comparisons containing LGALS1 gene expression by exposure/outcome. The comparisons in each use case were then interrogated for expression of 115 LGALS1 experimentally validated interactor genes from Integrated Interactions Database (IID), and 496 transcription factors predicted to regulate LGALS1 and its interactors from the Catalogue of Transcriptional Regulatory Interactions database (Catrin). Interactor genes and transcription factors that appeared across comparisons were then analyzed using PathDIP and hypergeometric tests to assess enriched pathways among the LGALS1 interactome in each use case. IFTA, interstitial fibrosis and tubular atrophy; IID, Integrated Interactions Database; Catrin, Catalogue of Transcriptional Regulatory Interactions.

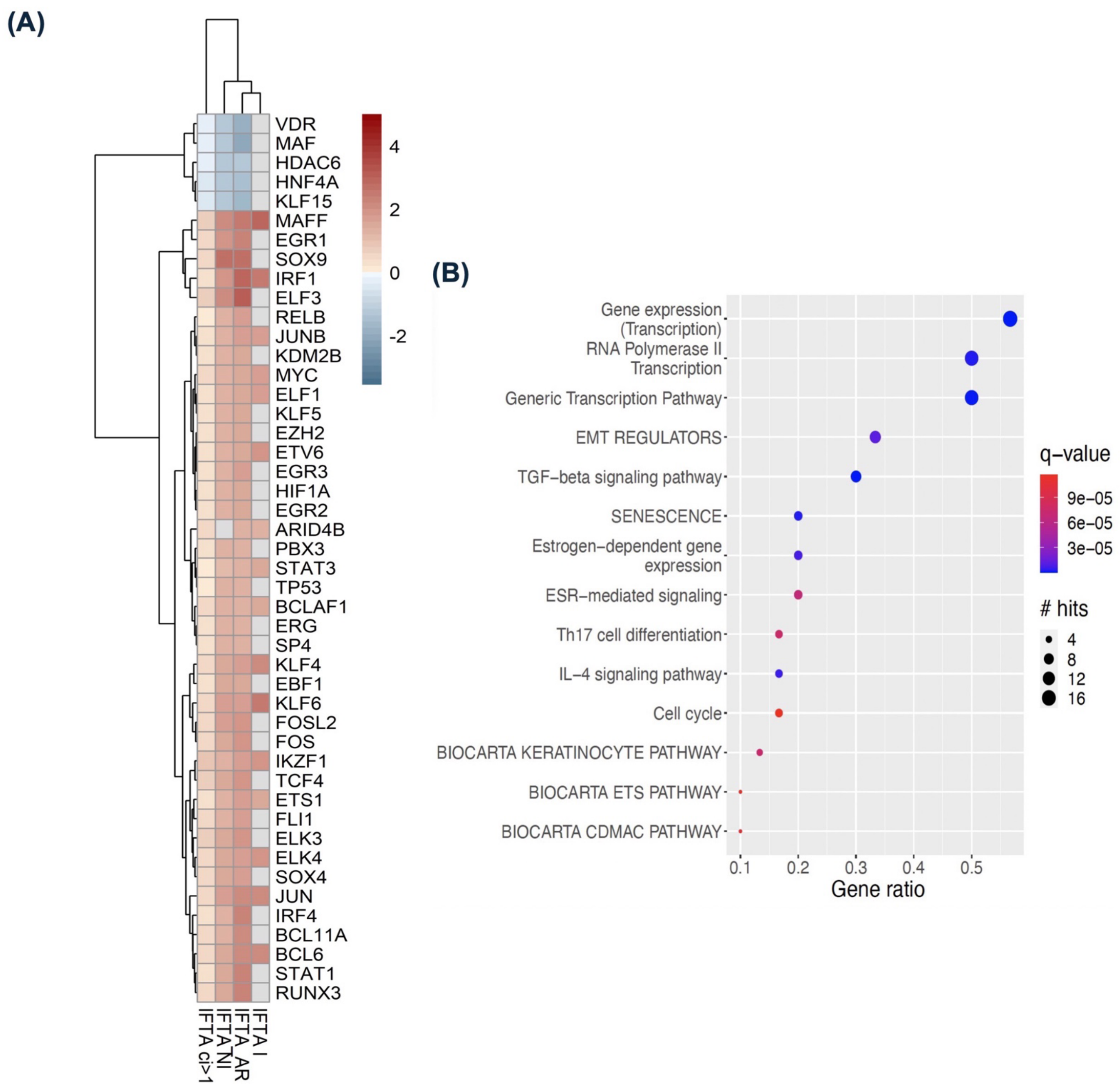

Supplementary Figure 5. LGALS1 transcription factor expression in the kidney allografts with interstitial fibrosis and tubular atrophy (IFTA) integrating data from Venner et al., 2016 and Modena et al., 2016 (36, 37). **(A)** Heatmap showing 46 LGALS1 transcription factor genes expressed in at least 3 comparisons, relative to normally functioning kidney transplants or kidney transplants with mild/no IFTA (ci</=1). Red indicates high expression and blue indicates low expression, relative to pre-transplantation or stable graft biopsies. **(B)** Top 15 pathways enriched (FDR:BH<0.01) for concordant transcription factors are shown. The size of the circle indicates the number of transcription factors in the pathway, and the colour indicates significance. IFTA, interstitial fibrosis and tubular atrophy; IFTA ci>1, interstitial fibrosis and tubular atrophy with at least moderate interstitial fibrosis score; IFTA-I, interstitial fibrosis and tubular atrophy with inflammation; IFTA-NI, interstitial fibrosis and tubular atrophy with no inflammation; IFTA-AR, interstitial fibrosis and tubular atrophy with acute rejection.

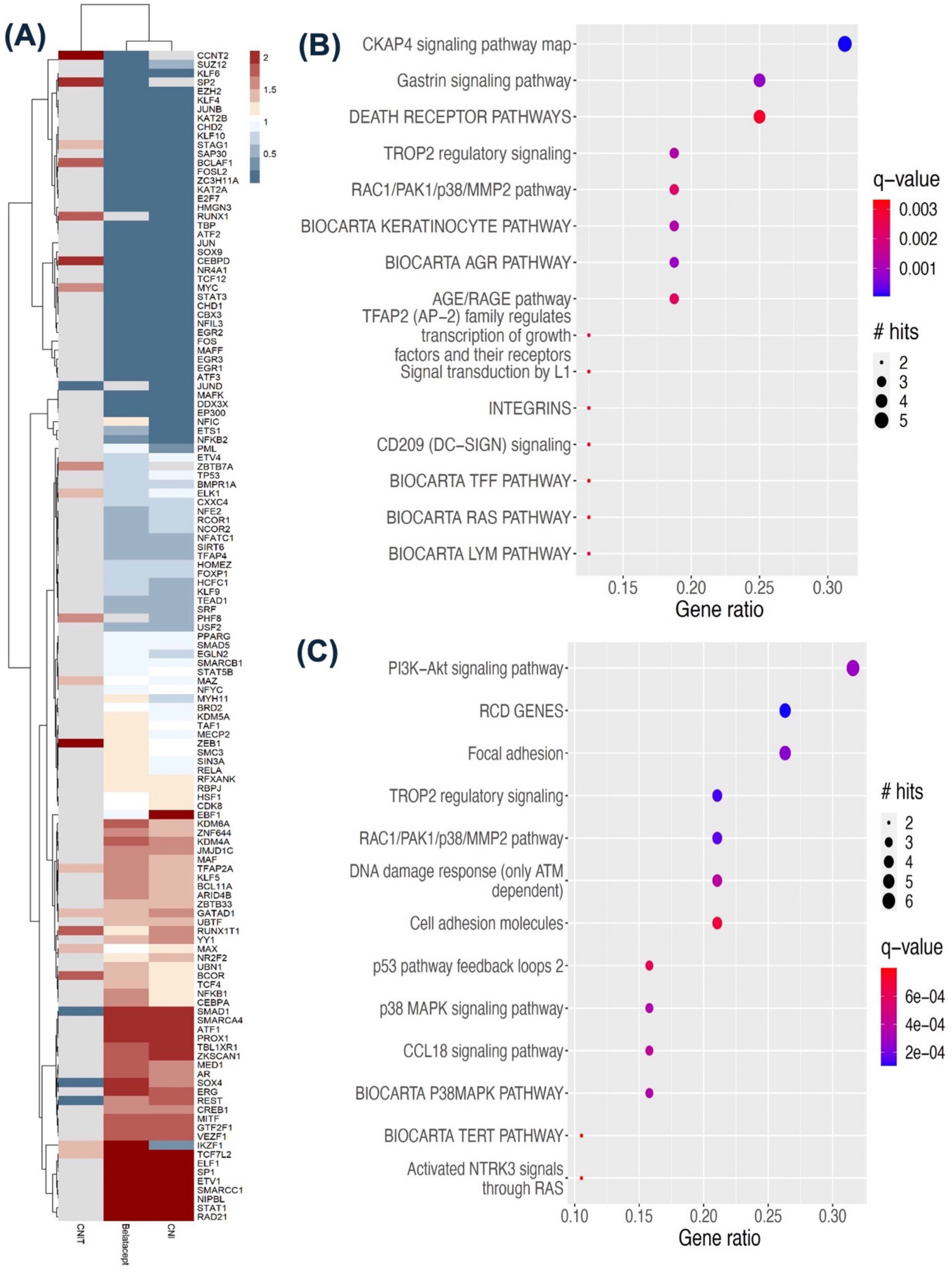

Supplementary Figure 6. LGALS1 and its interactome expression in the kidney, in the context of different immunosuppression regimens: CNI treatment or Belatacept, or CNI toxicity integrating data from Vitalone et al., 2014 and Rhone et al., 2021 (45, 46). (A**)** Heatmap shows 131 LGALS1 transcription factor genes expressed in at least 2 comparisons. Red indicates high expression and blue indicates low expression, relative to pre-transplantation or stable graft biopsies. Top 15 pathways significantly enriched (FDR:BH<0.01) among **(B)** concordant interactors or **(C)** discordant interactors are shown. The size of the circle indicates the number of LGALS1 interactors in the pathways and the colour indicates significance. CNI, calcineurin inhibitor; CNIT, calcineurin inhibitor toxicity.
